# Cryopreserved PM21-Particle-Expanded Natural Killer Cells Maintain Cytotoxicity and Effector Functions *In Vitro* and *In Vivo*

**DOI:** 10.3389/fimmu.2022.861681

**Published:** 2022-04-07

**Authors:** Jeremiah L. Oyer, Tayler J. Croom-Perez, Thomas A. Dieffenthaller, Liza D. Robles-Carillo, Sarah B. Gitto, Deborah A. Altomare, Alicja J. Copik

**Affiliations:** ^1^ Burnett School of Biomedical Sciences, College of Medicine, University of Central Florida, Orlando, FL, United States; ^2^ Department of Pathology and Laboratory Medicine, Abramson Cancer Center, Perelman School of Medicine, University of Pennsylvania, Philadelphia, PA, United States

**Keywords:** NK cells, NK cell therapy, cryopreservation, immunotherapy, cell therapy

## Abstract

There is a great interest in developing natural killer (NK) cells as adoptive cancer immunotherapy. For off-the-shelf approaches and to conduct multicenter clinical trials, cryopreserved NK cells are the preferred product. However, recent studies reported that cryopreservation of NK cells results in loss of cell motility and, as a consequence, cytotoxicity which limits the clinical utility of such products. This study assessed the impact of cryopreservation on the recovery and function of PM21-particle expanded NK cells (PM21-NK cells) as well as their antitumor activity *in vitro* using 2D and 3D cancer models and *in vivo* in ovarian cancer models, including patient-derived xenografts (PDX). Viable PM21-NK cells were consistently recovered from cryopreservation and overnight rest with a mean recovery of 73 ± 22% (N = 19). Thawed and rested NK cells maintained the expression of activating receptors when compared to expansion-matched fresh NK cells. Cryopreserved NK cells that were thawed and rested showed no decrease in cytotoxicity when co-incubated with tumor cells at varying effector-to-target (NK:T) ratios compared to expansion-matched fresh NK cells. Moreover, no differences in cytotoxicity were observed between expansion-matched cryopreserved and fresh NK cells in 3D models of tumor killing. These were analyzed by kinetic, live-cell imaging assays co-incubating NK cells with tumor spheroids. When exposed to tumor cells, or upon cytokine stimulation, cryopreserved NK cells that were thawed and rested showed no significant differences in surface expression of degranulation marker CD107a or intracellular expression of TNFα and IFNγ. *In vivo* antitumor activity was also assessed by measuring the extension of survival of SKOV-3-bearing NSG mice treated with fresh *vs.* cryopreserved NK cells. Cryopreserved NK cells caused a statistically significant survival extension of SKOV-3-bearing NSG mice that was comparable to that observed with fresh NK cells. Additionally, treatment of NSG mice bearing PDX tumor with cryopreserved PM21-NK cells resulted in nearly doubling of survival compared to untreated mice. These data suggest that PM21-NK cells can be cryopreserved and recovered efficiently without appreciable loss of viability or activity while retaining effector function both *in vitro* and *in vivo*. These findings support the use of cryopreserved PM21-NK cells as a cancer immunotherapy treatment.

## Introduction

Cellular immunotherapy is an emerging treatment modality for cancer, and therapies using CAR-T cells have been highly successful and gained recent FDA approval (1, 2). While T-cell-based therapy has been a major focus of immuno-oncology, it is known that dysfunction and suppression of the innate immune response can occur in cancer patients and strategies to restore these defects have the potential to greatly improve outcomes. Natural killer (NK) cells are a key component of the innate immune system and are cytotoxic effector cells that have an inherent ability to recognize virally infected, stressed, or cancerous cells without prior sensitization or antigen presentation [reviewed in (3)]. Given their potent antitumor response combined with their low risk for cytokine release syndrome (CRS) and graft-vs.-host disease (GVHD) using allogeneic sources, much effort has been put in recent years to develop methods to use adoptive NK cells, including CAR-NK cells, in immunotherapeutic treatments for cancer [reviewed in (4, 5)].

Preclinical studies using purified NK cells have demonstrated the therapeutic potential for many cancer types. Improvements in sources for NK cells and *ex vivo* expansion and activation methods [reviewed in (6)] have driven forward the ability to test the clinical efficacy of NK cell therapy; however, testing has been limited to pilot studies and early-phase clinical trials utilizing fresh NK cells at academic institutions with access to laboratories operating under Good Manufacturing Practice (GMP) to manufacture the NK cell product. Alternatively, centralized institutions for processing and manufacturing NK cells under GMP conditions remote from the clinic setting could be utilized, such as the National Heart, Lung, and Blood Institute (NHLBI)-sponsored Production Assistance for Cellular Therapies (PACT) program particularly if cryopreserved NK cells could be used (7–9). Thus, cryopreservation of NK cell products would greatly improve the availability for extensive clinical trials and potential future therapeutic use.

Historically, NK cells have been challenging to cryopreserve without loss of viability or function and initial clinical trials using cryopreserved NK cells showed poor *in vivo* survival and function (10–13). Several factors could influence how amenable NK cells are to cryopreservation, including expansion and stimulation methods, culturing conditions, and freezing medium. Optimizing methods of cryopreservation of different *ex vivo* expanded NK cells would greatly benefit their validation for use in adoptive cellular therapy. Expansion/activation protocols intended for clinical use should allow for cryopreservation of cells with high post-thaw recovery of viable cells that retain their function and antitumor activity. The objective of this study was to determine if PM21-NK cells, NK cells expanded *ex vivo* using a feeder-free particle-based approach, can be cryopreserved and retain their antitumor functions *in vitro* and *in vivo*. PM21-NK cells were expanded using plasma membrane particles prepared from the K562-mb21-41BBL cell line. PM-particle expansion of NK cells has been compared to conventional expansion using live feeder cells, and the rate and overall extent of expansion were comparable (14). Also, the antitumor efficacy of expanded NK cells was comparable between feeder cells and particle-expanded cells derived from the same cells (15). PM21-NK cells have been previously shown to be highly cytotoxic, and PM21 particles can further expand them *in vivo* with biodistribution into bone marrow and major organs (14, 16). In this study, PM21-NK cells were cryopreserved followed by in-depth characterization and their antitumor capacity compared to fresh expansion-matched control PM21-NK cells. Cryopreserved PM21-NK cells were found to be phenotypically similar to fresh PM21-NK cells, maintain cytotoxic functions *in vitro*, and persist, and have antitumor effects *in vivo*. This study provides evidence that PM21-NK cells can be developed as a clinical-grade NK cell product that can be cryopreserved with the potential for use in a wide range of broad clinical applications.

## Materials and Methods

### Cell Culture

Discarded buffy coats (leukocyte source) from de-identified, healthy donors were purchased from a local blood bank (OneBlood) and were used as source of NK cells for experiments. Peripheral blood mononuclear cells (PBMCs) were separated by density using Ficoll-Paque PLUS density gradient medium (GE Healthcare, Chicago, IL, USA). PBMCs were aliquoted and cryopreserved prior to use in experiments or expansion. NK cells were expanded using PM21 particles as previously described (16). Briefly, whole PBMCs or T-cell-depleted PBMCs (EasySep CD3 Positive Selection Kit; StemCell Technologies, Vancouver, Canada) were cultured for 13–14 days with 100 U/mL IL-2 (PeproTech, Cranbury, NJ, USA) and 200-µg/mL PM21 particles in SCGM media (CellGenix GmbH, Freiburg im Breisgau, Germany) supplemented with 10% FBS, 2 mM GlutaMAX. CSTX-002 cells (K562 cell line expressing 41BBL and membrane-bound IL-21) used for preparation of PM21 particles were provided by Kiadis Pharma, a Sanofi company. CSTX-002 cells were maintained in RPMI + 10% FBS. K562 (ATCC Cat# HTB-77, RRID : CVCL_0532) and SKOV-3 (ATCC Cat# HTB-77, RRID : CVCL_0532) cells were maintained according to ATCC recommendations. K562-GFP-luc cells were generated *via* stable transduction using lentiviral particles generated in-house (Addgene, Watertown, MA, USA). SKOV-3-NLR and A549-NLR cells were generated through stable transduction using commercial NucLight Red Lentivirus (Sartorius, Göttingen, Germany). All cell lines were positively selected *via* puromycin selection followed by sorting on uniform positive populations (BD FACSAria II). All cells were maintained in a humidified atmosphere at 37°C supplemented with 5% (vol/vol) CO_2_ in air. For mouse experiments, SKOV-3-GFP-Luc (RRID : CVCL_JY94) from Cell Biolabs, Inc. (San Diego, CA, USA), were passaged once through NSG mice to increase their tumorigenicity and sorted based on GFP expression. Cell lines were routinely tested for mycoplasma (e-Myco Plus Mycoplasma PCR Detection Kit, Bulldog Bio, Inc., Portsmouth, NH, USA) and authenticated *via* Human STR Profiling (serviced by ATCC).

### NK Cell Cryopreservation

Cells were centrifuged and resuspended at a final concentration of 1–2.5 × 10^7^ cells/mL in 50% RPMI/40% FBS/10% DMSO or a FBS-free, GMP-compliant commercial cryomedia formulation. The cell suspension was aliquoted into Nunc CryoTube Vials (Thermo Fisher Scientific) and immediately transferred to a Mr. Frosty™ Freezing Container (Thermo Fisher Scientific) and placed into -80°C overnight. Cryovials were then stored in liquid nitrogen until use. Both cryomedia formulations supported the cryopreservation of NK cells that resulted in similar recovery from thaw of NK cells that maintained the expression of activation markers post-thaw and cytotoxicity (**Supplemental Figure 1**). *In vitro* experiments were performed with NK cells cryopreserved in the commercial media formulation, and *in vivo* experiments were performed with NK cells cryopreserved in 50% RPMI/40% FBS/10% DMSO.

### Thawing NK Cells

Vials were transferred from liquid nitrogen to a 37°C water bath. The cell suspension was then diluted dropwise 5-fold in RPMI media (Cytiva HyClone) + 10% FBS + 1% antibiotic (Gibco) and the number of cells counted *via* flow cytometry and compared to the number of cells cryopreserved to confirm no loss in viable cells. 1 × 10^7^ cells were transferred to a T75 flask and brought up to 10 mL with RPMI media + 10% FBS + 1% antibiotic + 100 U/mL IL-2 (PeproTech) and incubated in a humidified atmosphere at 37°C supplemented with 5% (vol/vol) CO_2_ in air for 16 h. To calculate the percent recovery after an overnight rest, the total number of viable cells after an overnight rest was divided by 1 × 10^7^. For assays comparing fresh, 1-h post-thaw, and 16-h post-thaw NK cells, fresh samples were maintained in culture while donor- and expansion-matched NK cells from the same culture were cryopreserved on day 13 of the expansion and stored in liquid nitrogen for at least 1 h. The 16-h post-thaw samples were thawed on day 13 and rested for 16 h. The 1-h post-thaw samples were thawed on day 14, rested for 1 h, followed by assays collectively done on the fresh, 1-h post-thaw, and 16-h post-thaw samples.

### Flow Cytometry

The following pre-conjugated antibodies were used for flow cytometry: CD56-PE (clone 5.1H11, BioLegend Cat# 362508, RRID : AB_2563925), CD56-APC-Fire™750 (clone NCAM, BioLegend Cat# 362554, RRID : AB_2572105), CD56-AF647 (clone 5.1H11, BioLegend Cat# 362514, RRID : AB_2564087), CD3-APC (clone UCHT1, BioLegend Cat# 300439, RRID : AB_2562045), CD3-FITC (clone OKT3, BioLegend Cat# 317306, RRID : AB_571907), CD3-PerCP-eF710 (clone OKT3, Thermo Fisher Scientific Cat# 46-0037-42, RRID : AB_1834395), CD3-APC-Fire™750(clone OKT3, BioLegend Cat# 317352, RRID : AB_2800839), CD16-PeCy5 (clone 3G8, BioLegend Cat# 302010, RRID : AB_314210), NKG2D-APC (clone 1D11, BioLegend Cat# 320808, RRID : AB_492962), DNAM1-FITC (clone TX25, BioLegend Cat# 337104, RRID : AB_1236399), NKp30-PE (clone P30-15, BioLegend Cat# 325208, RRID : AB_756112), NKp46-PE/Dazzle™594 (clone 9E2, BioLegend Cat# 331930, RRID : AB_2566117), NKp44-PeCy7 (clone P44-8, BioLegend Cat# 325116, RRID : AB_2616754), CD69-PE (clone TP1.55.3, Beckman Coulter Cat# IM1943U, RRID : AB_2801272), Perforin-AF488 (clone DG9, BioLegend Cat# 308108, RRID : AB_493252), Granzyme B-AF532 (clone N4TL33, Thermo Fisher Scientific Cat# 58-8896-42, RRID : AB_2724390), IFNγ-PerCP-Cy5.5 (clone B27, BioLegend Cat# 506528, RRID : AB_2566187), TNFα-PE-Dazzle™594 (clone Mab11, BioLegend Cat# 502946, RRID : AB_2564173), CD107a-PE (clone H4A3, BioLegend Cat# 328608, RRID : AB_1186040), and CD45-eFluor™450 (clone 2D1, Thermo Fisher Scientific Cat# 48-9459-42, RRID : AB_1603240). DRAQ7™ (BioLegend Cat# 424001) was used as the cell viability dye. All samples were acquired on a CytoFlex S flow cytometer or Cytek Northern Lights Full Spectrum Flow Cytometer and analyzed using CytExpert (Beckman Coulter, Brea, CA, USA) or FlowJo software (RRID : SCR_008520 version 10.0.7).

### NK Cell Marker Expression Analysis

NK cells were isolated, expanded with PM21 particles, and frozen and thawed as described above. Fresh, 1-h post-thaw, and 16-h post-thaw NK cells were donor and expansion matched as described above and collectively analyzed for marker expression by flow cytometry using the pre-conjugated antibodies listed above. NK cells were selected for analysis based on gating for viable CD56^+^CD3^-^ cells.

### Cytotoxicity Assays

Cytotoxicity assays of NK cells utilized measurement of Annexin V or kinetic live-cell imaging. NK cells were isolated, expanded with PM21 particles, frozen, and thawed as described above. For Annexin V-based assays, target cells (K562-GFP-luc cells) were cultured at 0.6 × 10^6^ cells/mL alone (control wells) or cocultured with NK cells at indicated effector-to-target (E:T) ratios for 90 min in a humidified atmosphere at 37°C supplemented with 5% (vol/vol) CO2 in air. The cells were then centrifuged and resuspended in Annexin V labeling buffer containing Annexin V-eFluor™450 antibody (Thermo Fisher Scientific, Waltham, MA, USA, Cat# 88-8006-74, RRID : AB_2575164) and incubated for 15 min at 4°C prior to analysis by flow cytometry. The cytotoxicity was determined based on the absolute amount of Viable Target Cells (GFP^+^/Annexin V^-^) remaining in each well with effectors (VTC^E:T^) and referenced to average VTC in “target alone” control wells (VTC^T ctrl.^).


$$Cytotoxicity^{E:T}\left(\%\right)=\frac{1}{\left(\frac{VTC^{E:T}}{Avg\;VTC^{T\;ctrl}}\right)}\times 100$$

For kinetic live-cell imaging cytotoxicity assays, target cells (SKOV-3 NLR or A549 NLR) were cultured at 10,000 cells/well alone or cocultured with NK cells at indicated effector-to-target (E:T) ratios. Growth and fluorescence were monitored over time with an IncuCyte^®^ S3 System (Sartorius). For cytotoxicity against spheroids, target cells were seeded in round-bottom ultra-low-attachment plates (Corning, NY, USA, Ref#7007) 3 days prior to adding NK cells to form spheroids. Target cell growth/killing was monitored over time using red object count per well (ROC) to track tumor cell growth in 2D assays and total red object-integrated intensity (ROII) (RCU × μm^2^/Image) to track tumor spheroid growth in 3D assays. Relative expansion of the target cells alone or in the presence of NK cells (**Supplemental Figure 2**) was determined by normalizing ROC or ROII to the value at time 0 (ROC_t_/ROCt=0 or ROII_t_/ROIIt=0) when NK cells were initially added to the cultures to determine normalized ROC (nROC) or normalized ROII (nROII) (17). Cytotoxicity (%) was then determined based on the following equations. 


$$2D\;Cytotoxicity^{E:T}\left(\%\right)=\left(1-\left(\frac{nROC^{E:T}}{nROC^{T}}\right)\right)\times 100$$

$$3D\;Cytotoxicity^{E:T}\left(\%\right)=\left(1-\left(\frac{nROII^{E:T}}{nROII^{T}}\right)\right)\times 100$$

Concentration-dependent cytotoxicity curves were also plotted.

### IFNγ and TNFα Expression, and Degranulation

NK cells were stimulated with vehicle, target cells (K562), cytokines (10 ng/mL IL-12, 50 ng/mL IL-18, 100 ng/mL IL-15), or target cells + cytokines in the presence of 3 µg/mL brefeldin A (eBioscience, San Diego, CA, USA) and BD GolgiStop™ (BD Biosciences, Franklin Lakes, NJ, USA) for 4 h. Samples were then stained with CD56 and CD3 antibodies, fixed and permeabilized (eBioscience IC Fixation and Permeabilization buffers), and probed with dye-conjugated antibodies for IFNγ, TNFα, and CD107a followed by analysis using flow cytometry.

### Mouse Models

NSG (NOD-scid IL-2Rγnull, BCBC Cat# 4142, RRID : BCBC_4142) mice were purchased from The Jackson Laboratory (Bar Harbor, ME, USA) and then bred in-house. Female 8- to 12-week-old mice were used for all *in vivo* experiments. Mice were housed and handled in accordance with protocols approved by the University of Central Florida Institutional Animal Care and Use Committee, an Association for Assessment and Accreditation of Laboratory Animal Care International (AAALAC) accredited facility.

For the anti-SKOV-3 efficacy of fresh or frozen PM21-NK cells *in vivo* experiment, 1 × 10^6^ SKOV-3-GPF-luc cells were injected to the intraperitoneal (*i.p.*) cavity of NSG mice and allowed to seed for 4 days. Mice were then treated as specified in the figure legends, with 2 doses of 1 × 10^7^ NK cells injected to the *i.p.* along with IL-2 (25,000 U, 3×/week). Mice were imaged on day 26 after the first NK-cell injection using the In-Vivo Xtreme II™ imager (Bruker Daltonik GmbH, Bremen, Germany) and checked periodically for health status.

For the PDX model, tumor tissue was collected from an ovarian cancer PDX model TM00324 NSG mouse that had PDX ovarian papillary serous adenocarcinoma fine-needle aspirate injected subcutaneously (The Jackson Laboratory, Bar Harbor, ME, USA). Minced tumor tissue was intraperitoneally injected into NSG mice. Spheroids were obtained from the ascites that developed in the *i.p.*-passaged (twice) tumors, and 20,000 spheroids were injected in suspension into NSG mice *i.p.* and allowed to seed for 3 days. Mice were then treated as specified in the figure legends, with 2 doses of 1 × 10^7^ frozen NK cells from two different donors injected to the *i.p.* along with IL-2 (25,000 U, 3×/week) and health monitored periodically. Abdominal wash was collected from a mouse treated with frozen NK cells, euthanized 64 days posttreatment.

### Statistical Analysis

Statistical analyses were performed using GraphPad Prism (RRID : SCR_002798, version 9). Pairwise comparisons were performed to determine individual p-values. The area under the curve was used to compare cytotoxicity curves and determine p-value by unpaired t-tests. Multiple-paired, two-tailed Student’s t-test was used to analyze cytokine secretion and marker expression. Survival analysis was performed using the Gehan–Breslow–Wilcoxon test to determine statistical significance. Values less than 0.05 were considered significant. p values are shown as * if p < 0.05, ** if p < 0.01, *** if p < 0.001, and **** if p < 0.0001.

## Results

### Particle-Expanded NK Cells Are Viable After Cryopreservation, Thaw, and Rest

**Figure 1:**
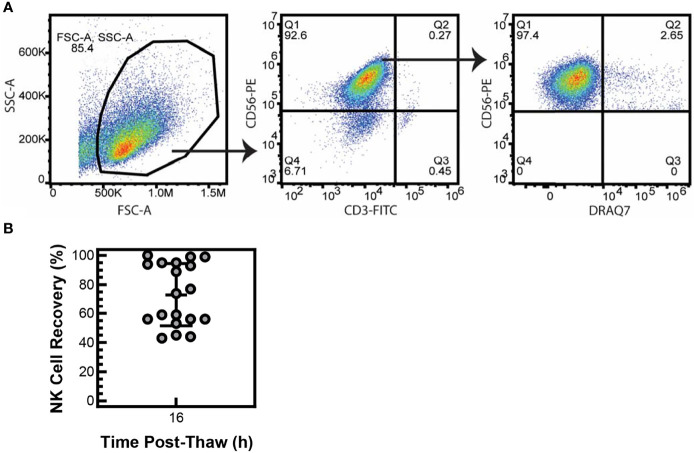
NK cells are viable after cryopreservation. NK cells were expanded with PM21 particles (PM21-NK cells) from T-cell-depleted PBMCs obtained from multiple donors (N = 19). Cells were cryopreserved and stored in liquid nitrogen until use. Frozen PM21-NK cells were thawed and counted for number of viable NK cells, and no loss of NK cells was seen. Representative flow cytometry dot plots gating for viable (Draq7^-^) NK (CD3^-^, CD56^+^) cells immediately post-thaw are shown **(A)**. Thawed NK cells were placed in media at 1 × 10^7^ of viable cells and rested overnight at 37°C. After 16 h, the total number of viable NK cells was counted. Percent recovery at 16 h post-thaw was calculated by dividing the total number of viable cells, remaining after 16 h rest, by 1 × 10^7^. Mean recovery was 73% ± 22% **(B)**. Data are represented as a scatter plot with an error bar representing standard deviation.

To determine if particle-expanded NK cells are viable after cryopreservation, NK cells were expanded with PM21-particles from T-cell-depleted PBMCs for 14 days (PM21-NK cells) and then cryopreserved and stored in liquid nitrogen. Cryopreserved PM21-NK cells were thawed and counted by flow cytometry, and gating on NK (identified as CD56^+^/CD3^-^) cells that are viable (identified as DRAQ7^-^) showed high (>95%) initial viability (**Figure 1A**) with no loss in the number of cells compared to the number of cells that were cryopreserved (data not shown). The viability shortly post-thaw may not reflect the actual health of the cells, and greater cell loss often occurs within 16 h post-thaw. To confirm that these viable NK cells can be recovered after overnight incubations, 1 × 10^7^ thawed viable NK cells were placed in media + 100 U/mL IL-2 and rested overnight at 37°C. After 16 h, the total number of viable NK cells was counted by flow cytometry. NK cells expanded from 19 different PBMC donors had a mean recovery of 73 ± 22% of the viable NK cells (**Figure 1B**). This indicates that immediately post-thaw, the cell viability of particle-expanded NK cells is not affected by cryopreservation and most of the cells can be consistently recovered, even after 16 h of rest.

### NK Cells Maintain Activating Receptor Expression After Cryopreservation

**Figure 2:**
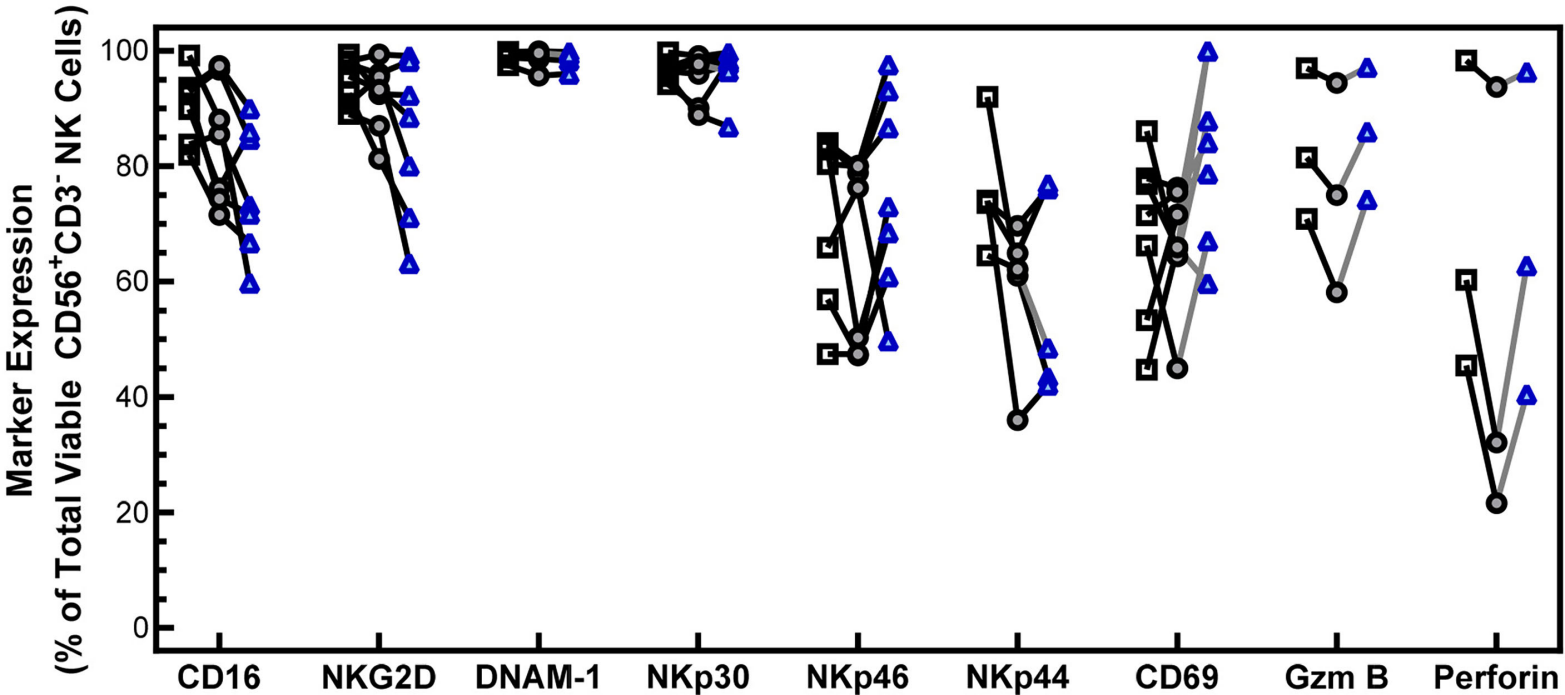
NK cells maintain the expression of activating receptors after cryopreservation. PM21-NK cells expanded from T-cell-depleted PBMCs were obtained from multiple donors (N = 3–7). Cells were cryopreserved while donor-matched fresh PM21-NK cells (black squares) were maintained in culture. Frozen PM21-NK cells were thawed and rested 16 h the day before analysis (blue triangles) or thawed and rested for 1 h on the day of analysis (gray circles). Expression of NK cell activating receptors CD16, NKG2D, DNAM-1, NKp30, NKp46, NKp44, as well as CD69, granzyme B, and perforin was determined by flow cytometry. No statistically significant difference was seen in the expression of activating receptors either 1 h immediately post-thaw or after a 16-h rest in donor and expansion-matched PM21-NK cells (N = 5–7 donors). Data are presented as a scatter plot with each point representing the average of two technical replicates with donor-pair lines. No statistical significance was determined by multiple paired t-tests with a threshold for significance at p < 0.05.

NK cell function and cytotoxicity rely in part on the expression of activating receptors. To confirm that cryopreservation of PM21-NK cells has no effect on the levels of activating receptors, the expression of 6 activating receptors (CD16, NKG2D, DNAM1, NKp30, NKp46, and NKp44) was analyzed 1 h and 16 h post-thaw on CD56^+^/CD3^-^ NK cells by flow cytometry and compared to fresh donor and expansion-matched PM21-NK cells (**Figure 2**). While there is a trend for decreased expression of CD16 and NKG2D post-thaw, no statistical significance was indicated in the expression of activating receptors either 1 h immediately post-thaw or after a 16-h rest in donor- and expansion-matched PM21-NK cells (N = 5–7 donors). Other markers for NK-cell activation and cytotoxic function were also assessed, and despite a trend for decrease in expression of activation markers CD69, granzyme, and perforin 1 h post-thaw, these were not statistically significant and their expression was recovered after 16 h of rest (**Figure 2**) (N = 3–7 donors). These results indicate that cryopreservation of PM21-NK cells does not affect the expression of activation markers.

### NK Cells Maintain Antitumor Response After Cryopreservation

**Figure 3:**
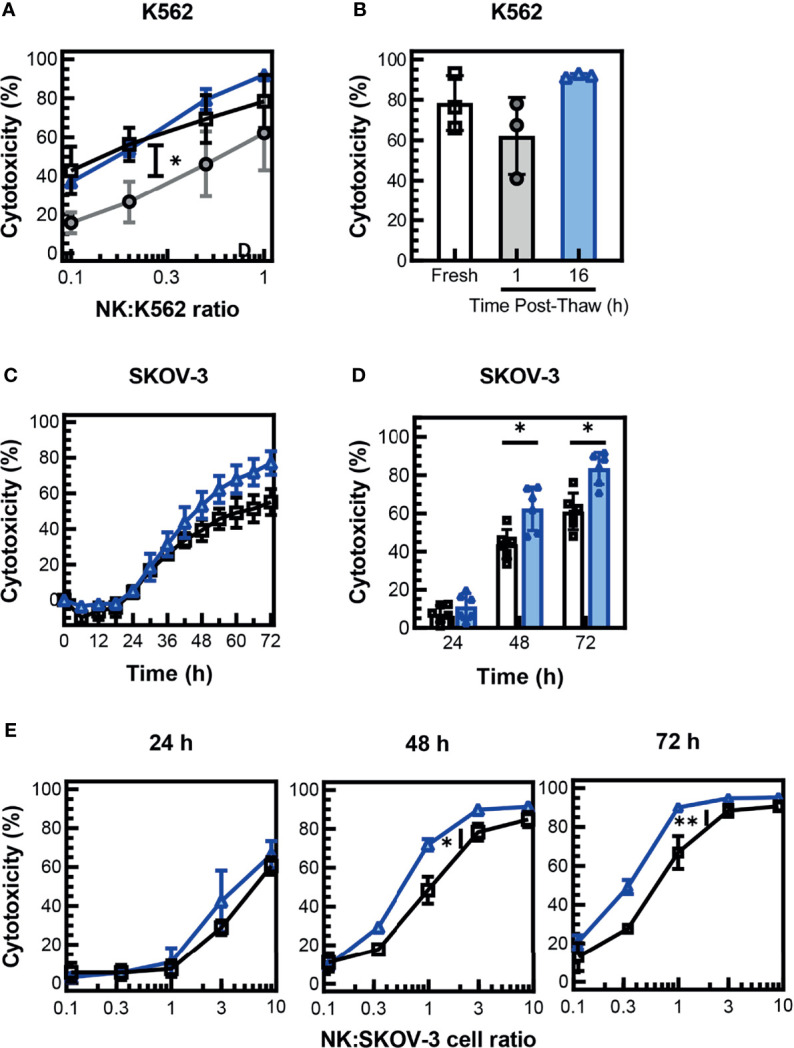
NK cells maintain cytotoxicity against tumor cells after cryopreservation. PM21-NK cells were expanded from T-cell-depleted PBMCs obtained from multiple donors (N = 2–3). Cells were cryopreserved while donor-matched fresh PM21-NK cells (black squares) were maintained in culture. Frozen NK cells were thawed and rested 16 h the day before analysis (blue triangles) or thawed and rested 1 h on the day of analysis (gray circles). Fresh or thawed NK cells were added to K562-GFPLuc cells at the indicated NK:K562 ratio **(A)** or at a ratio of 1:1 **(B)** and co-incubated for 60 min to measure cytotoxicity using Annexin V assay. Cryopreserved PM21-NK cells were less cytotoxic against K562 cells 1 h post-thaw compared to fresh PM21-NK cells but recovered cytotoxicity when rested 16 h post-thaw (N = 3 donors). **(A, B)**. Cryopreserved PM21-NK cells that were rested 16 h post-thaw (blue triangles), killed SKOV-3 cells similarly or better compared to matched fresh PM21-NK cells (black squares) (one representative donor in triplicate) **(C)**. Cryopreserved PM21-NK cells that were rested 16 h post-thaw (blue triangles), killed SKOV-3 cells similarly or better compared to matched fresh PM21-NK cells (black squares) at a 1:1 NK : SKOV-3 ratio (N = 2 in triplicate). **(D)** Concentration-dependent cytotoxicity curves were generated by measuring cytotoxicity at the indicated time-points of the cytotoxicity assay at multiple NK : SKOV-3 cell ratios **(E)**. Cryopreserved PM21-NK cells rested for 16 h post-thaw had comparable or increased cytotoxicity compared to donor and expansion-matched fresh PM21-NK cells (N = 2 in triplicate) **(E)**. Data are presented as scatter plots or bar graphs with error bars representing standard deviation. Statistical significance was determined by multiple paired t-tests. For cytotoxicity plots, the area under the curve was measured and then statistical significance was determined by unpaired t-tests. p values are shown as *p < 0.05, **p < 0.01.

Assessment of post-thaw expression of activating receptors on cryopreserved PM21-NK cells showed a trend for decreased expression of major activating receptor NKG2D, although not statistically significant. To assess if this change in expression affects the antitumor activity of the NK cells, cytotoxicity of the cryopreserved PM21-NK cells was measured 1 and 16 h post-thaw and compared to donor- and expansion-matched fresh PM21-NK cells. Concentration-dependent cytotoxicity curves were generated by measuring cytotoxicity against K562 cells at multiple NK:K562 cell ratios using Annexin V assays and were used to assess NK cells’ ability to kill tumor targets. Cryopreserved PM21-NK cells rested for 1 h were less cytotoxic compared to fresh cells as demonstrated by a statistically significant decrease in the area under the cytotoxicity curve (-21 ± 9 % ∙ ratio for 1 h vs. fresh, N = 3 donors, p = 0.03); however, cytotoxicity was recovered 16 h post-thaw (+6 ± 5 % ∙ ratio, N = 3) (**Figure 3A**). Cryopreserved PM21-NK cells rested for 1 h post-thaw killed less K562 cells than fresh NK cells (62% ± 19% compared to 78% ± 14% cytotoxicity) but recovered cytotoxicity when rested 16 h post-thaw (92% ± 1% cytotoxicity, N = 3 at 1:1 NK:K562) (**Figure 3B**). To confirm that cryopreserved PM21-NK cells maintain cytotoxicity against other cancer cell targets, cytotoxicity against SKOV-3 ovarian cancer cells was determined by kinetic live-cell imaging assay where cytotoxicity against SKOV-3 cells was monitored over time. Cryopreserved PM21-NK cells rested 16 h post-thaw were as cytotoxic as fresh donor- and expansion-matched PM21-NK cells against SKOV-3 cells at a 1:1 NK : SKOV-3 ratio (**Figure 3C**). Cryopreserved PM21-NK cells that were rested 16 h post-thaw killed SKOV-3 cells similarly or better when compared to matched fresh samples (mean cytotoxicity 11% ± 7% vs. 7% ± 4%, N = 2 at 24 h; 62% ± 11% vs. 44% ± 8% p = 0.02 at 48 h; 84% ± 8% vs. 61% ± 10%, N = 2, p = 0.01 for cryopreserved vs. fresh, respectively) (**Figure 3D**). Concentration-dependent cytotoxicity curves were generated by measuring cytotoxicity at multiple NK : SKOV-3 cell ratios. Cryopreserved PM21-NK cells rested for 16 h post-thaw had as good or better cytotoxicity as compared to donor- and expansion-matched fresh PM21-NK cells (N = 2) (**Figure 3E**).

**Figure 4:**
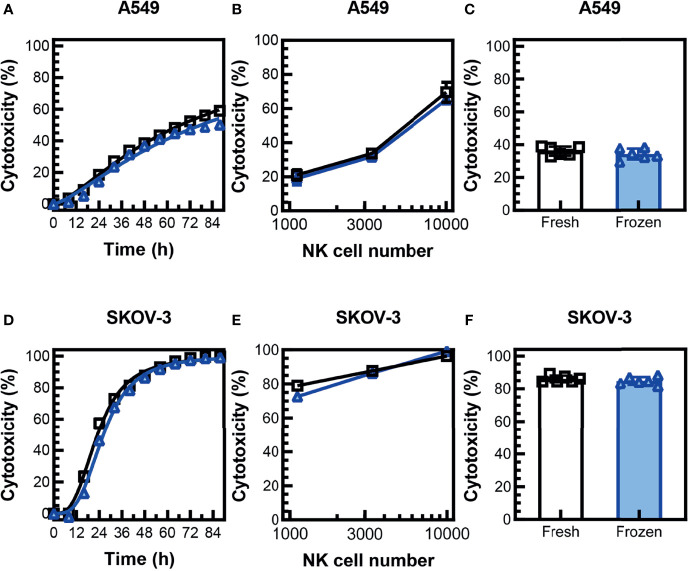
Cryopreserved NK cells maintain cytotoxicity in 3D tumor cell spheroid models. PM21-NK cells were expanded from T-cell-depleted PBMCs obtained from two donors. Cells were cryopreserved while donor-matched fresh PM21-NK cells were maintained in culture. Frozen NK cells were thawed and rested 16 h the day before analysis. NK-cell cytotoxicity was determined by kinetic live-cell imaging assay. Cryopreserved PM21-NK cells (blue triangles) were as efficacious at killing as fresh donor and expansion-matched PM21-NK cells (black squares) when 3,300 NK cells were cocultured with A549 spheroids **(A)** or SKOV-3 spheroids **(D)** (one representative donor in triplicate). Concentration-dependent cytotoxicity curves were also generated, and no significant difference between cryopreserved PM21-NK cells or fresh PM21-NK cells was found after 48 h of coculture with A549 spheroids **(B)** or SKOV-3 spheroids **(E)** (one representative donor in triplicate). Cryopreserved PM21-NK cells killed similarly A549 **(C)** or SKOV-3 **(F)** spheroids compared to matched fresh PM21-NK cells (3,300 NK/spheroid, N = 2 donors, in triplicate). Data are presented as scatter plots or bar graphs with error bars representing standard deviation. No statistical significance was determined by two-way ANOVA or multiple t-tests. For cytotoxicity time course plots, the area under the curve was measured and then statistical significance was determined by unpaired t-tests.

Previous studies have found that NK cells expanded with irradiated K562-expressing 41BBL and membrane-bound IL-21 (K562-mbIL15-41BBL) feeder cells had a significant decrease in cytotoxicity in a 3D environment after cryopreservation (18). To determine if cryopreserved PM21-NK cells retain the ability to induce cell lysis in a 3D tumor cell model, cytotoxicity against SKOV-3 ovarian and A549 lung cancer cell spheroids was monitored over time by kinetic live-cell imaging assay. Cryopreserved PM21-NK cells rested for 16 h post-thaw were as efficacious at killing as fresh donor- and expansion-matched PM21-NK cells when 3,300 NK cells were cocultured with A549 (**Figure 4A** **)** or SKOV-3 (**Figure 4D**) spheroids. Concentration-dependent cytotoxicity curves were also generated, and no significant difference between cryopreserved PM21-NK cells rested for 16 h post-thaw or fresh PM21-NK cells was found after 48 h of coculture with A549 spheroids (**Figure 4B**) or SKOV-3 spheroids (**Figure 4E**) (N = 2). Cryopreserved PM21-NK cells rested for 16 h post-thaw killed similarly A549 (**Figure 4C**) or SKOV-3 (**Figure 4F**) spheroids compared to matched fresh samples (mean cytotoxicity at 48 h: 34% ± 3% vs. 36% ± 3% for A549 spheroids; 85% ± 2% vs. 86% ± 2% for SKOV-3 spheroids, for cryopreserved vs. fresh, respectively [3,300 NK/spheroid, (N = 2)].

**Figure 5:**
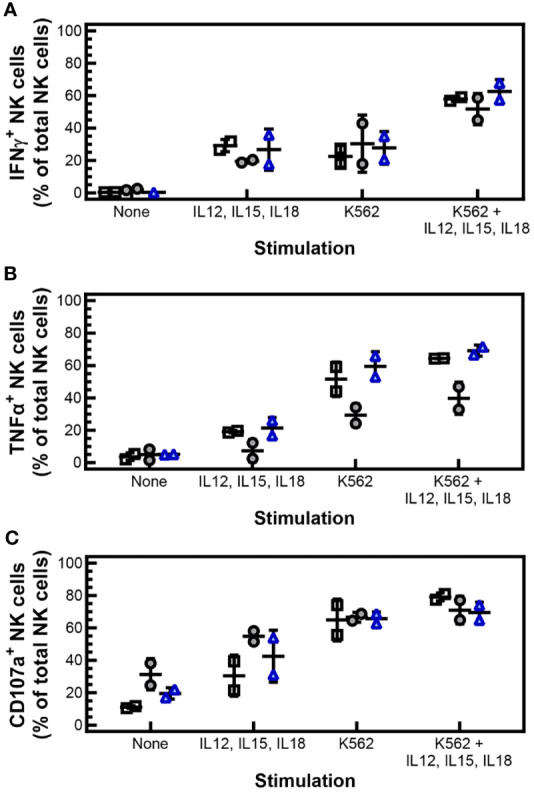
Cryopreserved, stimulated NK cells maintain IFNγ and TNFα expression, and degranulation. PM21-NK cells were expanded from T-cell-depleted PBMCs obtained from two donors. Cells were cryopreserved while donor-matched fresh PM21-NK cells were maintained in culture. Cryopreserved PM21-NK cells were thawed the day before analysis and rested 16 h or thawed on the day of analysis and rested 1 h. Intracellular expression of IFNγ and TNFα was measured in response to cytokine stimulation or engagement of tumor cells. Cryopreserved PM21-NK cells or donor and expansion-matched fresh PM21-NK cells were incubated with vehicle, cytokines (10 ng/mL IL-12, 100 ng/mL IL-15, 50 ng/mL IL-18), K562 cells, or K562 cells and cytokines in the presence of brefeldin A and BD GolgiStop™ to allow for intracellular accumulation and detection of cytokines by flow cytometry. Stimulation with cytokines, K562 cells, or both increased the fraction of NK cells expressing IFNγ compared to unstimulated cells similarly for cryopreserved PM21-NK cells rested for 1 h (gray circles) or 16 h post-thaw (blue triangles) compared to matched fresh PM21-NK cells (black squares) **(A)**. Expression of TNFα decreased 1 h post-thaw (gray circles) compared to fresh PM21-NK cells (black squares). This decrease in expression, however, was not statistically significant. TNFα expression recovered with a 16-h post-thaw rest (blue triangles) **(B)**. No significant difference in expression of surface CD107a was observed between cryopreserved or fresh donor and expansion-matched PM21-NK cells **(C)**. Data are presented as scatter plots with each point representing the average of two technical replicates with error bars representing standard deviation (N = 2). No statistical significance was determined by multiple paired t-tests with a threshold for significance at p < 0.05.

To further characterize the antitumor response of cryopreserved PM21-NK cells, intracellular expression’ of IFNγ and TNFα was measured in response to cytokine stimulation or engagement of tumor cells. Cryopreserved PM21-NK cells or donor and expansion-matched fresh PM21-NK cells were incubated with vehicle, cytokines (IL-12, IL-15, IL-18), K562 cells, or K562 cells and cytokines in the presence of brefeldin A and BD GolgiStop™ to allow for intracellular accumulation and detection of cytokines by flow cytometry. Stimulation with cytokines, K562 cells, or both, increased the fraction of NK cells expressing IFNγ compared to unstimulated cells, similar to what was measured for cryopreserved PM21-NK cells rested for 1 or 16 h post-thaw compared to matched fresh PM21-NK cells (**Figure 5A**). The expression of TNFα in cryopreserved PM21-NK cells decreased 1 h post-thaw compared to fresh cells, from 19% ± 1% to 7% ± 7% for cytokine stimulation, 52% ± 11% to 29% ± 7% for K562 stimulation, and 64% ± 1% to 40% ± 10% for stimulation with both cytokines and K562 cells (N = 2). This decrease in expression, however, was not statistically significant, and TNFα expression recovered after 16 h post-thaw rest [21% ± 6% for cytokine stimulation, 59% ± 9% for K562 stimulation, and 69% ± 3% for stimulation with both (**Figure 5B**) (N = 2)]. Expression of surface CD107a, a marker for degranulation, was also measured and no significant difference in expression was observed between cryopreserved or fresh PM21-NK cells (**Figure 5C**).

Overall, these data show that cryopreserved PM21-NK cells rested 16 h have the ability to kill tumor cells and secrete cytokines and degranulate upon stimulation, suggesting they maintain their capacity for an antitumor response.

### Cryopreserved PM21-NK Cells Are Efficacious *In Vivo*

**Figure 6:**
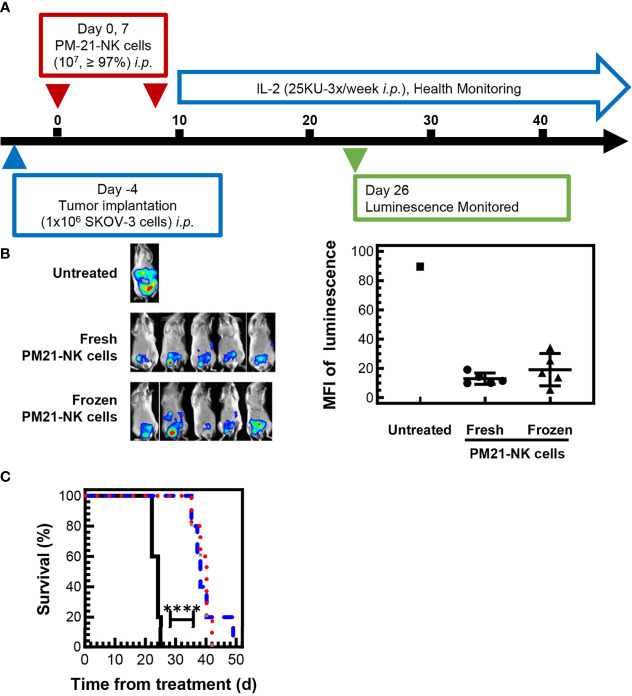
Cryopreserved NK cells maintain antitumor effect in an *in vivo* mouse model. 1 × 10^6^ SKOV-3-GFP-luc cells were injected to the intraperitoneal (*i.p.*) cavity of NSG (NOD-scid IL-2Rγnull) female mice and allowed to seed for 4 days. Mice were then treated with 2 doses of either fresh or cryopreserved NK cells that were thawed and directly injected *i.p.* along with IL-2 (25,000 U, 3×/week). Mice were imaged 26 days after the first NK cell injection using In-Vivo Xtreme II imager and checked periodically for health status. A schematic of the experiment is shown **(A)**. Day 26 *in vivo* images **(B)** and quantification of luminescence **(C)** showed that mice treated with either frozen NK cells (triangles) or fresh NK cells (circles) had lower and comparable tumor burden based on the luminescence quantification compared to the signal measured in the only remaining untreated mouse (square). Mice injected with frozen NK cells (red dotted line) or fresh NK cells (blue dashed line) had a similar significant increase in survival compared to untreated mice (black solid line). Statistical significance was determined by Survival Curve comparison using the Gehan–Breslow–Wilcoxon test.

**Figure 7:**
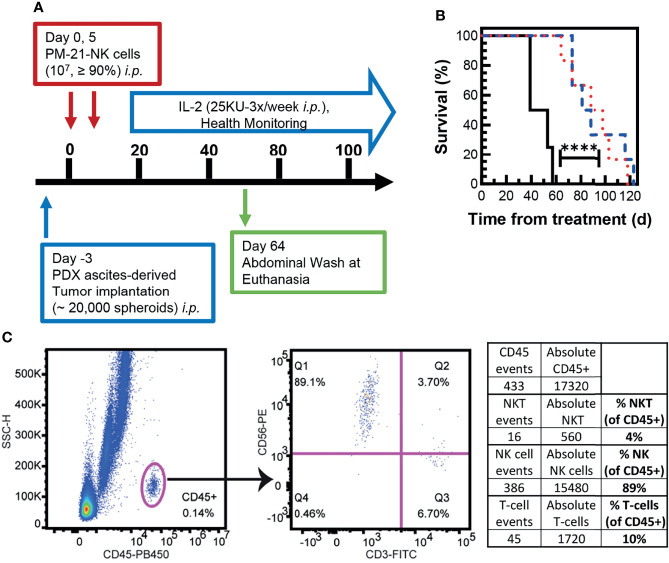
Cryopreserved NK cells exert antitumor effect in an *in vivo* PDX mouse model. Ascites were collected from an NSG mouse that had PDX tumor injected intraperitoneally. Spheroids were obtained from the ascites, and 20,000 spheroids were injected in suspension back to the intraperitoneal (*i.p.*) cavity of female NSG mice and allowed to seed for 3 days. Mice were then treated with 2 doses of cryopreserved NK cells (1 × 10^7^/dose, days 0 and 5) from two donors, which were thawed and directly injected *i.p.* along with IL-2 (25,000 U, 3×/week). Health was monitored periodically, and abdominal wash was collected from one mouse treated with frozen NK cells euthanized 64 days posttreatment. A schematic of the experiment is shown **(A)**. Mice injected with frozen NK cells from donor 1 (blue dashed line) and donor 2 (red dotted line) had a significant increase in survival compared to untreated mice (black solid line) **(B)**. Flow cytometry dot plots are shown for gating on CD45+ cells in the abdominal wash of a treated mouse euthanized on day 64 posttreatment **(C)**. The percentages of hNKT (CD3^+^, CD56^+^), hNK (CD3^-^,CD56^+^), and hT cells (CD3^+^) were determined in the hCD45^+^ population. Statistical significance was determined by comparing survival curves using the Gehan–Breslow–Wilcoxon test.

To test the efficacy of cryopreserved PM21-NK cells *in vivo*, an NSG (NOD-scid IL-2Rγnull) mouse model with SKOV-3-GFP-Luc ovarian cancer cells injected to the *i.p.* cavity was used to compare the antitumor activity of cryopreserved and fresh PM21-NK cells. Cryopreserved PM21-NK cells were injected immediately post-thaw to test their potential for clinical utility as an “off-the-shelf” product. After tumor cells were seeded for 4 days, mice were treated with 2 doses of 1 × 10^7^ either fresh or cryopreserved PM21-NK cells immediately post-thaw, injected to the *i.p*. cavity on day 0 and day 7 along with IL-2 (25,000 U, 3×/week) as shown in the schematic in **Figure 6A**. Mice were imaged 26 days after the first NK-cell injection, and treatment with either cryopreserved or fresh PM21-NK cells resulted in similar decreased levels of luminescence (13 ± 4 MFI and 19 ± 11 MFI, respectively, compared to 90 MFI in the only remaining animal in the control, untreated group) (**Figure 6B**). Treatment with either cryopreserved or fresh PM21-NK cells resulted in a significant increase in survival compared to untreated mice: a 38-day median survival with PM21-NK cells and 40 days for cryopreserved PM21-NK cells as compared to 24 days for untreated animals (**Figure 6C**). Further *in vivo* experiments using a PDX mouse model demonstrated antitumor activity of cryopreserved PM21-NK cells. Two NK donors were used to determine differences between donor sources. For the PDX model, ascites were collected from an NSG (NOD-scid IL-2Rγnull) mouse that had minced PDX tumor tissue injected to the *i.p.* cavity. Spheroids were obtained from the ascites, and the suspension was injected again *i.p.* into NSG mice and allowed to seed for 3 days. Mice were then treated with 2 doses of 1 × 10^7^ cryopreserved PM21-NK cells from two different donors injected to the *i.p.* cavity immediately post-thaw along with IL-2 (25,000 U, 3×/week) as shown in the schematic in **Figure 7A**. Mice treated with cryopreserved PM21-NK cells had a significant increase in survival, 84-day median survival for donor 1, and 93-day median survival for donor 2, compared to untreated mice with a median survival of 46 days, and no statistical difference was observed between NK donors (**Figure 7B**). Abdominal wash collected from a mouse treated with cryopreserved PM21-NK cells from donor 2 was analyzed by flow cytometry for presence of NK cells. NK cells persisted in the *i.p.* cavity and could be detected 64 days posttreatment (**Figure 7C**). Collectively, these data show that cryopreserved PM21-NK cells persist and are efficacious in *in vivo* mouse models.

## Discussion

Immunotherapy has had dramatic success in the treatment of cancer and led to a paradigm shift in oncology. Adoptive cellular therapy is an emerging immunotherapy modality that has been successfully used alone and as an adjuvant treatment (19). Current clinical trials of cellular therapies are using autologous or allogeneic cells, either unmodified or exogenously engineered, to increase their function or specificity. Chimeric antigen receptor (CAR)-T cells represent a highly successful engineering approach to target T cells to specific tumor antigens. In 2017, the US FDA approved the first gene therapy, KYMRIAH CAR-T cell therapy, for treatment of acute lymphoblastic leukemia, and since then four additional autologous CAR-T cell immunotherapies for the treatment of cancer have been approved (2). There are, however, known side effects of using T-cell therapy such as CRS and macrophage activation syndrome (MAS)/hemophagocytic lymphohistiocytosis (HLH) (20–22), and given other immune cell types play a critical role in the immune response to cancer, alternative cellular therapies are being explored with the goal of decreasing toxicity while retaining the efficacy of the CAR cell product.

Recent studies have demonstrated the importance of functional NK cells for the success of immunotherapies and they have potential for an “off-the-shelf” efficacious adoptive cell therapy with an improved safety profile [Reviewed in (23)]. These innate cytotoxic effector cells have the ability to recognize tumor cells with decreased antigen expression and pose a low likelihood of severe adverse effects like CRS. Preclinical studies using purified NK cells have demonstrated the therapeutic potential for many cancer types. However, testing the clinical efficacy for NK cell therapy thus far has been limited to pilot studies and early-phase clinical trials relying on fresh products prepared on-site (24). Historically, NK cells have been challenging to cryopreserve without loss of viability or function and initial clinical trials using cryopreserved NK cells showed poor *in vivo* survival and function (10–13). In a small clinical trial (of 8 patients) testing the safety, persistence, and activity of expanded NK cells in patients with high-risk relapsing myeloma, NK cells expanded from PBMCs with K562 cells expressing IL-15 and 4-1BBL were shipped overnight cryopreserved from the production site to the trial site (25). While the viability immediately post-thaw was high, thawed expanded NK cells failed to lyse K562 cells unless incubated overnight with a high concentration of IL-2 and recovery was extremely poor after overnight incubation, and therefore switch was made after the first 3 patients to fresh NK cell products (25). This is a clear example that robust reliable methods for cryopreservation of NK cells are needed to provide the ability to produce consistent large-scale products that can expand options for clinical application, including extensive quality control testing, multiple rounds of infusions, and multisite studies. It is therefore critical to determine methods for stimulation that result in a final product that is amenable for cryopreservation. Also, cryopreservation protocols that maintain not only the viability of NK cells but also antitumor effects are critical.

This study demonstrated that particle-expanded PM21-NK cells can be cryopreserved and maintain cytotoxicity *in vitro* and are efficacious *in vivo*. Cryopreserved PM21-NK cells were viable after thawing with consistent recovery of most of thawed cryopreserved PM21-NK cells even after 16 h of rest in the presence of low concentration of IL-2. The presence of low-concentration IL-2 (100 U/mL) was not essential for NK-cell persistence after 16 h of rest (**Supplemental Figure 3A**). This positively contrasts with previous reports that found loss of viable expanded NK cells after 16 h in culture, even in the presence of IL-2 (26, 27).

Compared to early trials of NK-cell cryopreservation, it has been suggested that recent methods using mbIL21 for NK-cell expansion could have altered NK-cell metabolic or antiapoptotic mechanisms that promote their survival after cryopreservation (28), which could be contributing to retention of viability of cryopreserved PM21-NK cells. Recently, a phase 1/2 study for patients with relapsed/refractory AML to determine the safety and feasibility of NK-cell therapy following chemotherapy was conducted. This study used multiple infusions of cryopreserved NK cells expanded with feeder cells genetically modified to express mbIL21 and 4-1BBL and found that NK cells generated in this way could be safely administered, and clinical outcomes were encouraging indicating adequate activity of the cryopreserved product (29).

Expression of activating receptors in NK cells is critical for their cytotoxic response. Cryopreserved PM21-NK cells maintain the expression of a large set of activating receptors, measured at 1 and 16 h post-thaw. Previous reports have shown decreased NKG2D and CD16 (18, 27, 30). A trend for decreased expression of both of these receptors was seen for cryopreserved PM21-NK cells from some donors; however, the difference was not statistically significant. Other markers for NK-cell activation and cytotoxic function CD69, granzyme, and perforin were decreased 1 h post-thaw but recovered after 16 h rest, suggesting recovery and activation of the NK cells. Furthermore, the potential antitumor activity of the cryopreserved NK cells was confirmed by comparable ability to kill various tumor cell lines and similar levels of IFNγ, TNFα, and CD107a produced after stimulation with cytokines and/or K562 cells.

Previously, it was reported that NK cells expanded using EBV-LCL feeder cells maintained excellent viability after being frozen and thawed, but the cytolytic capacity of the NK cells to kill K562 cells was greatly reduced compared to fresh NK cells (30). In this study, PM21-NK cells had reduced cytotoxicity 1 h post-thaw but fully recovered cytotoxicity at 16 h post-thaw across the range of tested NK:K562 cell ratios. The ability of PM21-NK cells to maintain cytotoxic function additionally was not dependent on the presence of IL-2 in the media during the rest period (**Supplemental Figure 3B**). Cytotoxicity against K562 cells was measured in a short 90-min assay, and previous studies found that cryopreserved and thawed NK cells expanded with K562-mbIL15-41BBL cells were cytotoxic *via* antibody-dependent cell-mediated cytotoxicity (ADCC) immediately post-thaw in a 4-h toxicity assay, but in a 24-h cytotoxicity assay ADCC occurred only in the first 4 h then tumor outgrowth occurred (27). To assess the cytotoxic ability of cryopreserved PM21-NK cells in longer time-course assays, kinetic live-cell imaging was conducted over 72 h during coculture with SKOV-3 target cells using multiple NK : SKOV-3 ratios and no decrease in cytotoxicity was observed, confirming that PM21-NK cells retain their cytotoxic activity after cryopreservation.

In a recent study, decreased cytotoxic function of cryopreserved *ex vivo*-expanded NK cells was attributed to impairment of NK-cell motility (18). Cryopreserved PM21-NK cells and fresh PM21-NK cells had similar abilities to kill tumor spheroids based on kinetic, live cell-imaging assay, suggesting that cryopreserved PM21-NK cells maintain function in a 3D environment. Ultimately, *in vivo* experiments treating mice with cryopreserved PM21-NK cells that were administered immediately post-thaw confirmed their ability to function in a 3D environment where motility is required. CD56 plays a critical role in NK-cell mobility and is involved in mediating NK-cell development and maturation (31). CD56 expression could be a predictor of cell health and successful cryopreservation (32).

Persistence of cryopreserved NK cells is another important aspect for developing successful cell-based therapeutics. Previously, cGMP-grade NK cells expanded with K562 cells expressing mbIL-15 and 41BBL were cryopreserved, thawed, and immediately infused into NOD/IL-2Rγc/Rag (NSG) mice that were given 250 cGy total body irradiation by X-ray. After 7 days, few cryopreserved NK cells persisted compared to mice infused with fresh NK cells. Overnight culture in IL-2 containing media only slightly increased the number of NK cells found on day 7 (26). In the current study, cryopreserved NK cells injected immediately post-thaw persisted in the *i.p.* cavity 64 days after treatment, suggesting cryopreserved cells are not only viable and functional but can persist in the tumor microenvironment *in vivo*. Taken together, even though there is some loss of cells after cryopreservation, the retained or even enhanced cytotoxicity post-thaw together with long persistence of cells resulted in strong antitumor activity *in vivo*, which was comparable to that measured with fresh cells.

This study has demonstrated that PM21-NK cells can be cryopreserved and recovered maintaining potency and antitumor effects *in vitro* and *in vivo*. In-depth characterization and comparison to matched fresh samples of this cryopreserved NK cell product established its feasibility for clinical use. This report in combination with recent studies that showed promising results in the cryopreservation of *ex vivo* expanded NK cells that maintain their function post-thaw (33–35) lay the groundwork for developing a clinical grade NK-cell product with potentially broad clinical applications.

## Data Availability Statement

The original contributions presented in the study are included in the article/**Supplementary Material**. Further inquiries can be directed to the corresponding author.

## Ethics Statement

The animal study was reviewed and approved by the University of Central Florida Institutional Animal Care and Use Committee, an Association for Assessment and Accreditation of Laboratory Animal Care International (AAALAC)-accredited facility.

## Author Contributions

JO: conceptualization, experimentation, data analysis, review and editing of the manuscript. TC-P: data analysis, figure preparation, writing, review, and editing of the manuscript. TD: experimentation, data analysis, review and editing of the manuscript. LR-C: experimentation, data analysis, review and editing of the manuscript. SG: mouse model experimentation. DA: mouse model administration and experimentation, review of the manuscript. AC: conceptualization, funding acquisition, project administration, resources, supervision, writing—review and editing. All authors contributed to the article and approved the submitted version.

## Funding

We thank the FL DOH James and Ester King Program (Grant No. 9JK04), the Guillot-Henley Family AML Research Fund in loving memory of William L. Guillot, the University of Central Florida Preeminent Postdoctoral Program, and Kiadis, a Sanofi Company, for the funds that supported this research.

## Conflict of Interest

AC: licensed IP to, consultancy and research support from Kiadis Pharma, a Sanofi company; JLO: licensed IP to, consultancy with Kiadis Pharma, a Sanofi company; DA: licensed IP to Kiadis Pharma, a Sanofi company.

The remaining authors declare that the research was conducted in the absence of any commercial or financial relationships that could be construed as a potential conflict of interest.

## Publisher’s Note

All claims expressed in this article are solely those of the authors and do not necessarily represent those of their affiliated organizations, or those of the publisher, the editors and the reviewers. Any product that may be evaluated in this article, or claim that may be made by its manufacturer, is not guaranteed or endorsed by the publisher.

## Abbreviations

4-1BBL, 4-1BB ligand; ADCC, antibody-dependent cell-mediated cytotoxicity; AML, acute myeloid leukemia; CAR, chimeric antigen receptor; CD16, Fc fragment of IgG receptor IIIα CD107a; LAMP1, lysosomal-associated membrane protein 1; CRS, cytokine release syndrome; DNAM-1, CD226 molecule; GMP, Good Manufacturing Practice; GVHD, graft-*vs.*-host disease; HLH/MAS, macrophage activation syndrome and hemophagocytic lymphohistiocytosis; IFN, interferon; mbIL15, membrane-bound IL-15; mbIL21, membrane-bound IL-21; NHLBI, National Heart, Lung, and Blood Institute; NK cells, natural killer cells; NKG2D, KLRK1 killer cell lectin like receptor K1; NKp30, NCR3 natural cytotoxicity triggering receptor 3; NKp44, NCR2 natural cytotoxicity triggering receptor 2; NKp46, NCR1 natural cytotoxicity triggering receptor 1; PACT, production assistance for cellular therapies; PBMCs, peripheral blood mononuclear cells; PDX, patient-derived xenograft; PM21-particles, plasma membrane particles; TNF-α, tumor necrosis factor alpha.

## References

[1] Haydu JE Abramson JS. CAR T-Cell therapies in lymphoma: current landscape, ongoing investigations, and future directions. J Cancer Metastasis Treat (2021) 7:36–50. doi: 10.20517/2394-4722.2021.39

[2] U.S. Food & Drug Administration Office of Tissues and Advanced Therapies (OTAT). Approved Cellular and Gene Therapy Products 2021 (2021). Available at: https://www.fda.gov/vaccines-blood-biologics/cellular-gene-therapy-products/approved-cellular-and-gene-therapy-products.

[3] Farag SS Caligiuri MA. Human Natural Killer Cell Development and Biology. Blood Rev (2006) 20(3):123–37. doi: 10.1016/j.blre.2005.10.001 16364519

[4] Cheng M Chen Y Xiao W Sun R Tian Z. NK Cell-Based Immunotherapy for Malignant Diseases. Cell Mol Immunol (2013) 10(3):230–52. doi: 10.1038/cmi.2013.10 PMC4076738 23604045

[5] Myers JA Miller JS. Exploring the NK Cell Platform for Cancer Immunotherapy. Nat Rev Clin Oncol (2021) 18(2):85–100. doi: 10.1038/s41571-020-0426-7 32934330 PMC8316981

[6] Lamb MG Rangarajan HG Tullius BP Lee DA. Natural Killer Cell Therapy for Hematologic Malignancies: Successes, Challenges, and the Future. Stem Cell Res Ther (2021) 12(1):211. doi: 10.1186/s13287-021-02277-x 33766099 PMC7992329

[7] Reed W Noga SJ Gee AP Rooney CM Wagner JE McCullough J. Production Assistance for Cellular Therapies (PACT): Four-Year Experience From the United States National Heart, Lung, and Blood Institute (NHLBI) Contract Research Program in Cell and Tissue Therapies. Transfusion (2009) 49(4):786–96. doi: 10.1111/j.1537-2995.2008.02027.x PMC4165072 19170985

[8] Koepsell SA Miller JS McKenna DH Jr. Natural Killer Cells: A Review of Manufacturing and Clinical Utility. Transfusion (2013) 53(2):404–10. doi: 10.1111/j.1537-2995.2012.03724.x 22670662

[9] Rezvani K Rouce RH. The Application of Natural Killer Cell Immunotherapy for the Treatment of Cancer. Front Immunol (2015) 6:578. doi: 10.3389/fimmu.2015.00578 26635792 PMC4648067

[10] Voshol H Dullens HF Den Otter W Vliegenthart JF. Human Natural Killer Cells: A Convenient Purification Procedure and the Influence of Cryopreservation on Cytotoxic Activity. J Immunol Methods (1993) 165(1):21–30. doi: 10.1016/0022-1759(93)90102-d 8409465

[11] Lapteva N Durett AG Sun J Rollins LA Huye LL Fang J. Large-Scale *Ex Vivo* Expansion and Characterization of Natural Killer Cells for Clinical Applications. Cytotherapy (2012) 14(9):1131–43. doi: 10.3109/14653249.2012.700767 PMC4787300 22900959

[12] Dominguez E Lowdell MW Perez-Cruz I Madrigal A Cohen SB. Natural Killer Cell Function is Altered by Freezing in DMSO. Biochem Soc Trans (1997) 25(2):175S. doi: 10.1042/bst025175s 9191219

[13] Holubova M Miklikova M Leba M Georgiev D Jindra P Caprnda M. Cryopreserved NK Cells in the Treatment of Haematological Malignancies: Preclinical Study. J Cancer Res Clin Oncol (2016) 142(12):2561–7. doi: 10.1007/s00432-016-2247-8 PMC11819048 27614454

[14] Oyer JL Igarashi RY Kulikowski AR Colosimo DA Solh MM Zakari A. Generation of Highly Cytotoxic Natural Killer Cells for Treatment of Acute Myelogenous Leukemia Using a Feeder-Free, Particle-Based Approach. Biol Blood Marrow Transplant. (2015) 21(4):632–9. doi: 10.1016/j.bbmt.2014.12.037 25576425

[15] Oyer JL Gitto SB Altomare DA Copik AJ. PD-L1 Blockade Enhances Anti-Tumor Efficacy of NK Cells. Oncoimmunology (2018) 7(11):e1509819. doi: 10.1080/2162402X.2018.1509819 30377572 PMC6205063

[16] Oyer JL Pandey V Igarashi RY Somanchi SS Zakari A Solh M. Natural Killer Cells Stimulated With PM21 Particles Expand and Biodistribute *In Vivo*: Clinical Implications for Cancer Treatment. Cytotherapy. (2016) 18(5):653–63. doi: 10.1016/j.jcyt.2016.02.006 27059202

[17] Varudkar N Oyer JL Copik A Parks GD. Oncolytic Parainfluenza Virus Combines With NK Cells to Mediate Killing of Infected and non-Infected Lung Cancer Cells Within 3D Spheroids: Role of Type I and Type III Interferon Signaling. J Immunother Cancer. (2021) 9(6):e002373. doi: 10.1136/jitc-2021-002373 34172515 PMC8237729

[18] Mark C Czerwinski T Roessner S Mainka A Horsch F Heublein L. Cryopreservation Impairs 3-D Migration and Cytotoxicity of Natural Killer Cells. Nat Commun (2020) 11(1):5224. doi: 10.1038/s41467-020-19094-0 33067467 PMC7568558

[19] Waldman AD Fritz JM Lenardo MJ. A Guide to Cancer Immunotherapy: From T Cell Basic Science to Clinical Practice. Nat Rev mmunol (2020) 20(11):651–68. doi: 10.1038/s41577-020-0306-5 PMC7238960 32433532

[20] Zheng PP Kros JM Li J. Approved CAR T Cell Therapies: Ice Bucket Challenges on Glaring Safety Risks and Long-Term Impacts. Drug Discov Today (2018) 23(6):1175–82. doi: 10.1016/j.drudis.2018.02.012 29501911

[21] Hansen DK Dam M Faramand RG. Toxicities Associated With Adoptive Cellular Therapies. Best Pract Res Clin Haematol (2021) 34(3):101287. doi: 10.1016/j.beha.2021.101287 34625233

[22] Sandler RD Tattersall RS Schoemans H Greco R Badoglio M Labopin M. Diagnosis and Management of Secondary HLH/MAS Following HSCT and CAR-T Cell Therapy in Adults; A Review of the Literature and a Survey of Practice Within EBMT Centres on Behalf of the Autoimmune Diseases Working Party (ADWP) and Transplant Complications Working Party (TCWP). Front Immunol (2020) 11:524. doi: 10.3389/fimmu.2020.00524 32296434 PMC7137396

[23] Shaver KA Croom-Perez TJ Copik AJ. Natural Killer Cells: The Linchpin for Successful Cancer Immunotherapy. Front Immunol (2021) 12:679117. doi: 10.3389/fimmu.2021.679117 33995422 PMC8115550

[24] Liu E Marin D Banerjee P Macapinlac HA Thompson P Basar R. Use of CAR-Transduced Natural Killer Cells in CD19-Positive Lymphoid Tumors. New Engl J Med (2020) 382(6):545–53. doi: 10.1056/NEJMoa1910607 PMC7101242 32023374

[25] Szmania S Lapteva N Garg T Greenway A Lingo J Nair B. *Ex Vivo*-Expanded Natural Killer Cells Demonstrate Robust Proliferation *In Vivo* in High-Risk Relapsed Multiple Myeloma Patients. J Immunother. (2015) 38(1):24–36. doi: 10.1097/CJI.0000000000000059 25415285 PMC4352951

[26] Miller JS Rooney CM Curtsinger J McElmurry R McCullar V Verneris MR. Expansion and Homing of Adoptively Transferred Human Natural Killer Cells in Immunodeficient Mice Varies With Product Preparation and *In Vivo* Cytokine Administration: Implications for Clinical Therapy. Biol Blood Marrow Transplant. (2014) 20(8):1252–7. doi: 10.1016/j.bbmt.2014.05.004 PMC4099265 24816582

[27] Damodharan SN Walker KL Forsberg MH McDowell KA Bouchlaka MN Drier DA. Analysis of *Ex Vivo* Expanded and Activated Clinical-Grade Human NK Cells After Cryopreservation. Cytotherapy. (2020) 22(8):450–7. doi: 10.1016/j.jcyt.2020.05.001 PMC7387178 32536506

[28] Lee DA. Cellular Therapy: Adoptive Immunotherapy With Expanded Natural Killer Cells. Immunol Rev (2019) 290(1):85–99. doi: 10.1111/imr.12793 31355489

[29] Silla L Valim V Pezzi A da Silva M Wilke I Nobrega J. Adoptive Immunotherapy With Double-Bright (CD56(bright) /CD16(bright) Expanded Natural Killer Cells in Patients With Relapsed or Refractory Acute Myeloid Leukaemia: A Proof-of-Concept Study. Br J Haematol (2021) 195(5):710–21. doi: 10.1111/bjh.17751 34490616

[30] Berg M Lundqvist A McCoy P Jr. Samsel L Fan Y Tawab A. Clinical-Grade *Ex Vivo*-Expanded Human Natural Killer Cells Up-Regulate Activating Receptors and Death Receptor Ligands and Have Enhanced Cytolytic Activity Against Tumor Cells. Cytotherapy. (2009) 11(3):341–55. doi: 10.1080/14653240902807034 PMC2736058 19308771

[31] Mace EM Gunesch JT Dixon A Orange JS. Human NK Cell Development Requires CD56-Mediated Motility and Formation of the Developmental Synapse. Nat Commun (2016) 7:12171. doi: 10.1038/ncomms12171 27435370 PMC4961740

[32] Lugthart G van Ostaijen-ten Dam MM van Tol MJD Lankester AC Schilham MW. CD56dimCD16– NK Cell Phenotype can be Induced by Cryopreservation. Blood. (2015) 125(11):1842–3. doi: 10.1182/blood-2014-11-610311 25766566

[33] Jung D Baek YS Lee IJ Kim KY Jang H Hwang S. *Ex Vivo* Expanded Allogeneic Natural Killer Cells Have Potent Cytolytic Activity Against Cancer Cells Through Different Receptor-Ligand Interactions. J Exp Clin Cancer Res (2021) 40(1):333. doi: 10.1186/s13046-021-02089-0 34686187 PMC8539797

[34] Min B Choi H Her JH Jung MY Kim HJ Jung MY. Optimization of Large-Scale Expansion and Cryopreservation of Human Natural Killer Cells for Anti-Tumor Therapy. Immune Netw (2018) 18(4):e31. doi: 10.4110/in.2018.18.e31 30181919 PMC6117513

[35] Oh E Min B Li Y Lian C Hong J Park GM. Cryopreserved Human Natural Killer Cells Exhibit Potent Antitumor Efficacy Against Orthotopic Pancreatic Cancer Through Efficient Tumor-Homing and Cytolytic Ability (Running Title: Cryopreserved NK Cells Exhibit Antitumor Effect). Cancers (Basel) (2019) 11(7):966–86. doi: 10.3390/cancers11070966 PMC6678894 31324057

