# Supplementary material for: Cryopreserved PM21-Particle-Expanded Natural Killer Cells Maintain Cytotoxicity and Effector Functions In Vitro and In Vivo
Source: Front Immunol. 2022 Apr 7;13:861681. doi: 10.3389/fimmu.2022.861681 (PMC9022621; doi:10.3389/fimmu.2022.861681)
Supplement: Supplementary file 1 [file DataSheet_1.pdf]

Supplemental Figure 1. PM21-NK cells recover and are cytotoxic after cryopreservation with commercial cryomedia formulation or 50% RPMI/40% FBS/10% DMSO.

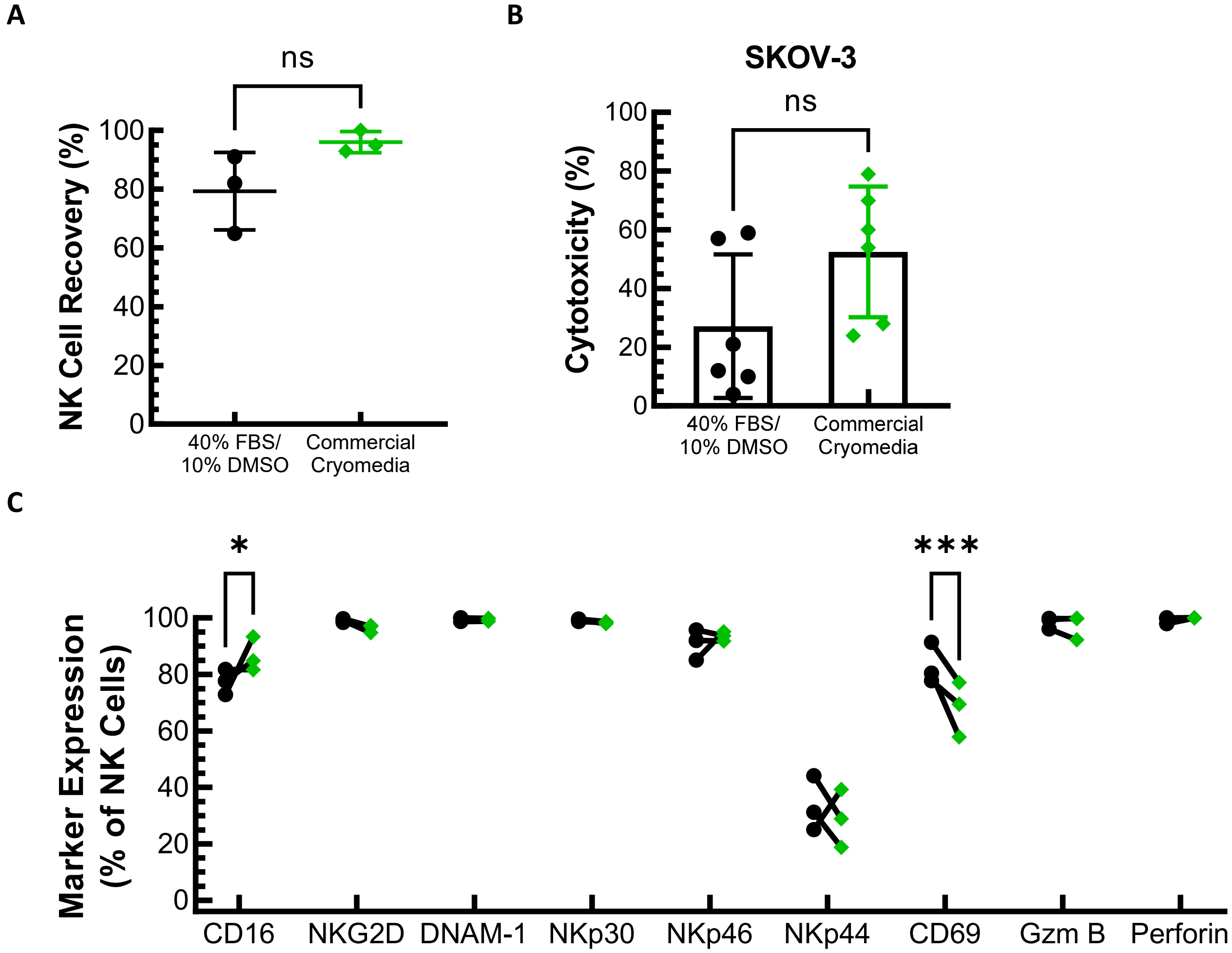

Supplemental Figure 1. PM21-NK cells recover and are cytotoxic after cryopreservation with commercial cryomedia formulation or 50% RPMI/40% FBS/10% DMSO. NK cells were expanded with PM21-particles (PM21-NK cells) from T cell-depleted PBMCs obtained from multiple donors (N = 3). Cells were cryopreserved with either commercial cryomedia (green diamonds) or 50% RPMI/40% FBS/10% DMSO (black circles). Thawed NK cells were placed in media at $4x10^6$ of viable cells and rested overnight at 37 °C. After 16 h the total number of viable NK cells was counted. Percent recovery at 16 h post-thaw was calculated by dividing the total number of viable cells, remaining after 16 h rest, by 4 x 10^6. No significant difference in mean recovery was seen between cryomedia formulations (**A**). A live-cell imaging cytotoxicity assay was used to show no difference in cytotoxic response of PM21-NK cells cryopreserved in two different media formulations against SKOV-3 cells after 20 h of co-culture at 4:1 NK:SKOV-3 ration (**B**). Expression of NK cell activating receptors CD16, NKG2D, DNAM-1, NKp30, NKp46, and NKp44, CD69, Granzyme B, and Perforin was determined by flow cytometry. No statistically significant difference was seen in the expression of activating receptors except CD16 and CD69 (**C**). Data are represented as a scatter plots with error bars representing standard deviation, scatter plots with each point representing the average of two technical replicates with donor-pair lines, or bar graphs with error bars representing standard deviation. Statistical significance was determined by multiple paired t-tests with a threshold for significance at $p < 0.05$.

Supplemental Figure 2. Relative Expansion of Target Cell in kinetic live-cell imaging cytotoxicity assays

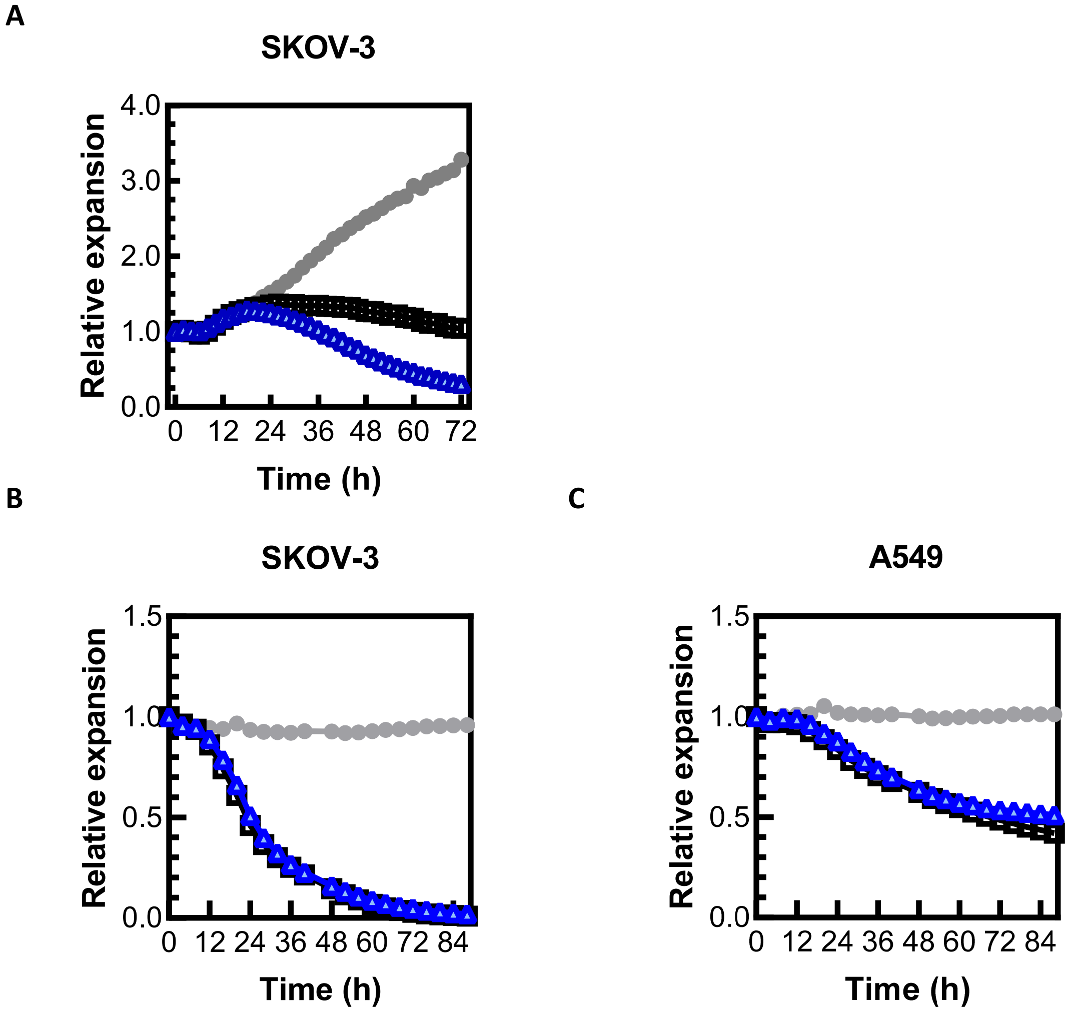

Supplemental Figure 2. Relative Expansion of Target Cell in kinetic live-cell imaging cytotoxicity assays. PM21-NK cells were expanded from T cell-depleted PBMCs obtained from multiple donors (N = 2). Cells were cryopreserved while donor matched fresh PM21-NK cells were maintained in culture. Frozen NK cells were thawed and rested 16 h the day before analysis. Fresh or cryopreserved PM21-NK cells were used in kinetic live-image cytotoxicity assays against 2D cultures of SKOV-3 (**A**) or spheroid cultures of SKOV-3 (**B**) or A549 (**C**). Data presented are the relative expansion of target cell alone (gray circles), target cells with fresh PM21-NK cells (black squares) or cryopreserved PM21-NK cells (blue triangles). Relative expansion of target cells co-cultured with PM21-NK cells compared to target cells alone was used to determine cytotoxicity over time.

Supplemental Figure 3. PM21-NK cells recover and are cytotoxic after cryopreservation with or without IL-2 present post-thaw.

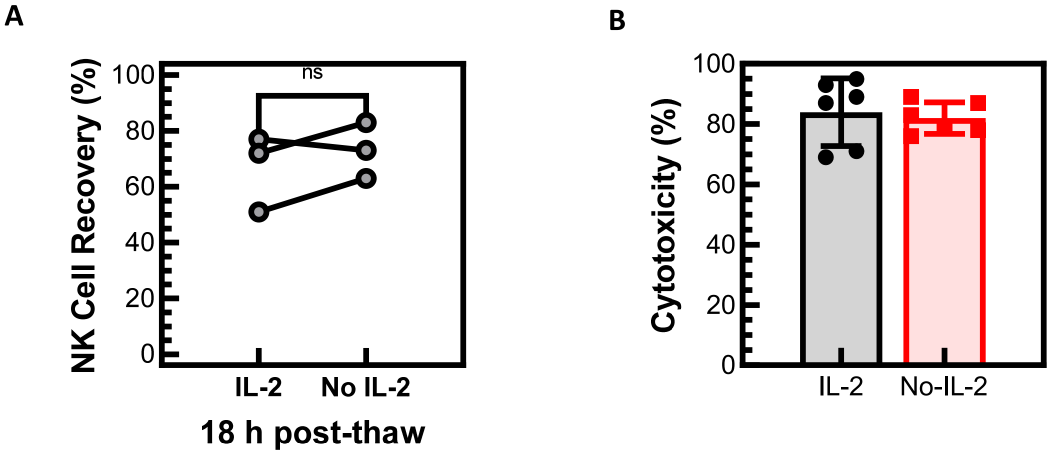

Supplemental Figure 3. PM21-NK cells recover and are cytotoxic after cryopreservation with or without IL-2 present post-thaw. NK cells were expanded with PM21-particles (PM21-NK cells) from T cell-depleted PBMCs obtained from multiple donors (N = 3). Cells were cryopreserved and stored in liquid nitrogen until use. Thawed NK cells were placed in media with or without 100 U/mL IL-2 at $1x10^7$ of viable cells and rested overnight at 37 °C. After 16 h the total number of viable NK cells was counted. Percent recovery at 18 h post-thaw was calculated by dividing the total number of viable cells, remaining after 18 h rest, by 1×10^7. Mean recovery was 67% ± 8% with IL-2 present and 73% ± 6% without IL-2 present (**A**). NK cells were added to K562-GFPLuc cells at a 1:1 NK:K562 ratio 18 h post-thaw and co-incubated for 90 min to measure cytotoxicity using Annexin V assay. No significant difference was observed in cytotoxicity against K562 cells with (black circles) or without (red squares) IL-2 present during 18 h rest post-thaw (**B**). Data are presented as scatter plots with donor-matched paired lines or bar graphs with error bars representing standard deviation. No statistical significance was determined by paired t-tests.
